# Correction to “The role of nurses and midwives in medical laboratory investigations in sub‐Saharan Africa”

**DOI:** 10.1002/hsr2.70070

**Published:** 2024-10-18

Window M, Nayupe SF, Msiska MT, Lucero‐Prisno DE. The role of nurses and midwives in medical laboratory investigations in sub‐Saharan Africa. *Health Sci Rep*. 2024;*7*(5):e2082. https://doi.org/10.1002/hsr2.2082

The correct Abstract should read as follows:

Across the globe, nurses and midwives play a crucial role in providing care to patients in healthcare facilities. They often contact the patient, providing direct care as directed by medical doctors or clinical officers. Traditionally, the role of nurses and midwives in the clinical diagnosis process is to coordinate the clinical diagnosis process—which includes laboratory diagnosis requests—from diagnosticians to the clinical laboratory. In these settings, these diagnosticians are general or specialist medical doctors. However, in some regions in sub‐Saharan Africa (SSA), nurses and midwives are primary diagnosticians in healthcare facilities. We present a perspective on the role of nurses and midwives in medical laboratory investigations in SSA. We highlight how, on top of nursing and midwifery roles, nurses take up the role of diagnosticians in facilities where doctors are few or are absent and what key issues are worth consideration. Furthermore, we present how efficient collaboration between nursing midwifery and medical laboratory diagnostic systems facilitates effective patient management. Emphasizing training on laboratory test utilization for nurses and midwives in SSA is vital for enhancing healthcare outcomes.

There was an omitted suffix “III” on Don Eliseo Lucero‐Prisno name. The correct name should read as “Don Eliseo Lucero‐Prisno III”

The correct affiliations of Don Eliseo Lucero‐Prisno III are as follows:

1. Department of Global Health and Development, Faculty of Public Health and Policy, London School of Hygiene and Tropical Medicine, London, UK
2. Office for Research, Innovation and Extension Services, Southern Leyte State University, Sogod, Southern Leyte, Philippines
3. Center for University Research, University of Makati, Makati City, Philippines

We apologize for this error.

